# Unveiling the fundamentals of two-phase axial-flow-induced vibrations of cantilever rods

**DOI:** 10.1038/s41598-026-35337-4

**Published:** 2026-01-13

**Authors:** Hao Li, Andrea Cioncolini, Hector Iacovides, William Benguigui, Mostafa R. A. Nabawy

**Affiliations:** 1 Department of Mechanical and Aerospace Engineering, The University of Manchester, Oxford Road, Manchester, M13 9PL UK; 2 School of Transportation Science and Engineering, Beihang University, Beijing, 100191 China; 3 Department of Mechanical Engineering (Robotics), Guangdong Technion - Israel Institute of Technology (GTIIT), 241 Daxue Road, Shantou, 515063 Guangdong China; 4 EDF R&D, Fluid Mechanic division, 6 Quai Watier, Chatou, 78400 France; 5 IMSIA, UMR EDF/CNRS/ENSTA 9219, Université Paris-Saclay, Palaiseau, France; 6 Aerospace Engineering Department, Faculty of Engineering, Cairo University, Giza, 12613 Egypt

**Keywords:** Engineering, Physics

## Abstract

Flow-induced vibrations (FIVs) in nuclear fuel assemblies can cause fretting wear and costly unplanned reactor outages, yet fundamental mechanistic understanding and predictive modelling of FIVs in gas-liquid flows remain hindered by the lack of non-intrusive diagnostic tools. Here, we introduce a Hall-effect-based electromagnetic sensing technique that, for the first time, enables comprehensive resolution of the axial-FIV dynamics of a cantilevered rod with different tip geometries over a range of air-water flow regimes. Our experiments reveal that increasing the void fraction amplifies chaotic vibrations while suppressing periodic oscillations, a transition driven by the increased intensity of stochastic-forcing induced by gas-liquid interactions and bubble impacts. As such, a dual-regime response emerges where vibration amplitudes increase at low Reynolds numbers but decrease/plateau at high Reynolds numbers. Strikingly, beyond a critical void fraction of 0.2, amplitudes converge across Reynolds numbers, signalling two-phase stochastic force dominance. Our findings elucidate the mechanistic competition between stochastic and periodic excitations in two-phase axial-FIVs with a simplified paradigmatic configuration that provides valuable preliminary information for nuclear reactor applications. The developed novel technique provides an enabling tool for real-time, non-intrusive FIV diagnostics, with potential applications extending beyond nuclear engineering.

**Supplementary Information:**

The online version contains supplementary material available at 10.1038/s41598-026-35337-4.

## Introduction

At present, there are over 400 nuclear power reactors operating worldwide, with a combined generation capacity of around 400 GWe that accounts for 10% of global electricity and 25% of the world’s low-carbon supply, making nuclear power the second-largest source of low-carbon electricity after hydroelectric power^1,2^. The world’s electricity demand is projected to surge by over 80% by 2050^3^, driven by industrial electrification^4–7^, electric vehicle diffusion^8–10^, increasing air conditioning^11–13^, and AI-driven data centres^14,15^. With 66 nuclear reactors currently under construction worldwide, 85 reactors planned, and an additional 344 reactor proposed^16^, nuclear power will remain pivotal to low-carbon electricity generation^17–19^. A representative example is China, currently the largest greenhouse gas emitter, which aims to peak emissions by 2030 and achieve carbon neutrality by 2060 while constructing 30 reactors, planning 36, and proposing 158 more^16^.

Most operational and upcoming reactors are water-cooled designs^20,21^, relying on fuel assemblies composed of slender zirconium-clad rods housing uranium dioxide pellets^22^, where the fission reaction takes place to generate heat^23^. These rods are cooled by axial water flow: pressurized water reactors (PWRs) operate at 15 MPa to maintain liquid coolant in the core, while boiling water reactors (BWRs) operate at 7 MPa, allowing in-core steam generation. Despite their near-continuous operation (interrupted only by 3-week refuelling/maintenance cycles), unplanned outages (costing €1M daily for a 1 GWe reactor) frequently stem from fuel rod failures caused by flow-induced vibrations (FIVs)^24,25^. Turbulence and unsteadiness in the coolant flows are the main sources of stochastic excitation that triggers fuel rod vibrations, in addition to periodic fluid forces caused by rod motion and amplified by tight core geometries. The resulting cyclic relative motion between the fuel rods and their support structures leads to rubbing, fretting wear, and eventually premature failures^26^.

The economic impact of unplanned outages has motivated extensive research into FIVs, with recent studies focusing on simplified cantilever rod configurations^27–33^ comprising a single cantilever rod confined inside a tube and exposed to an axial water flow. By combining design simplicity with rich and informative physics (the sources of excitation include turbulent buffeting, movement-induced excitation, and flow separation), cantilever rod configurations have become paradigmatic test cases that have been instrumental in clarifying the fundamental physics of axial-FIVs, and have also contributed high-resolution data for the development of numerical simulation models^34–39^. Specifically, theoretical models by Païdoussis^40^ predicted buckling and oscillatory instabilities in cantilevered rods, which were later validated experimentally^41^. Subsequent experimental studies revealed how the rod’s tip geometry and flow relative direction with respect to the rod’s boundary condition (‘*free-clamped*’ when the flow is directed from the rod free-end towards the rod clamped-end, vs. ‘*clamped-free*’ when the flow is directed the other way around) modulate vibration amplitudes via turbulence and motion-induced excitations^27,31–33,42^. For instance, a blunt tip configuration amplifies vibrations of free-clamped rods due to enhanced flow unsteadiness, while conical tip configurations are capable of suppressing them for the same boundary conditions^33^. In fact, turbulent buffeting and flow unsteadiness are both important contributors to the observed vibrations, necessitating the use of advanced turbulence models to enable accurate simulation of the problem^37–39^.

While extensive research has characterized cantilever rod axial-FIVs with single-phase flows, the mechanics underpinning FIVs with gas-liquid two-phase flows has been investigated to a lesser extent and remains poorly understood. Two-phase flows introduce complex fluid-structure interactions, absent in single-phase systems, modulated by the flow regimes (i.e., bubbly, slug, churn, and annular) associated with the corresponding homogeneous void fraction ($\:{\upalpha\:}$), defined as the ratio of gas to total superficial velocity^43,44^. Each flow regime is expected to alter the phase distribution, turbulence, and slip velocity, potentially reshaping the associated FIV excitation mechanisms^45^. For example, dispersed gas bubbles within bubbly flows can dampen rod vibrations, while chaotic interfacial interactions within slug or annular flows may amplify them. However, despite early flow-regime mappings^43,44,46^, no study has ever linked the observed flow patterns to FIV dynamics in axial flows.

Progress in cantilever rod two-phase FIVs has been hindered by measurement challenges: optical methods fail in two-phase flows due to bubble occlusion, while classical vibration sensors, e.g., accelerometers, are intrusive and unsuited for cantilever rod test pieces. To overcome these barriers, we are proposing a Hall-effect-based electromagnetic sensing technique, enabling non-intrusive, high-resolution vibration tracking in two-phase environments. As such, this work presents the first comprehensive experimental study on axial-FIV of cantilevered rods subjected to air-water flows, spanning single-phase to high-void-fraction two-phase flow regimes. Our experimental results correlate rod vibration dynamics with flow patterns, uncovering regime-specific flow excitation mechanisms and the impact of Reynolds number and void fraction on rod vibration amplitude and frequency. The present results advance the fundamental understanding of the physics of two-phase axial-FIVs and also establish a much-needed benchmark dataset for developing numerical simulation models. This is of immediate relevance for BWRs, the second most common nuclear reactor design after PWR, with 41 operational units and two more units under construction worldwide.

## Results and discussion

### Hall-effect measurement method for two-phase FIV diagnostics

**Fig. 1:**
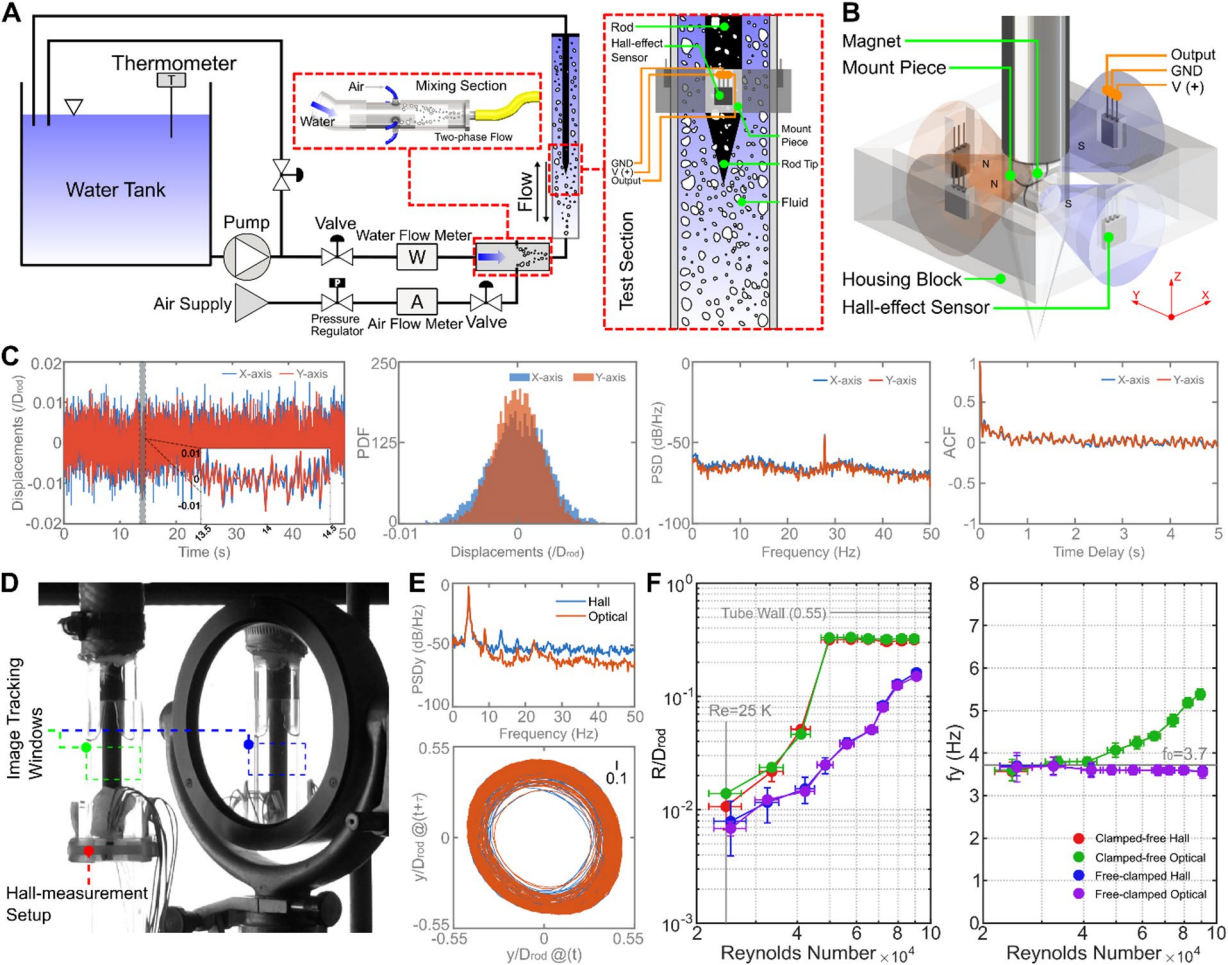
Experimental setup and Hall-effect system validation. (**A**) Schematic of the flow loop with insets showing the mixing and test sections, a conical tip geometry is used for this illustration (designs of blunt, hemispherical, and conical tips: Supplementary Material, Fig. S1). (**B**) Schematic of the Hall effect system assembly: A cylindrical mount piece, attached to the vibrating rod, embedded with 8 neodymium magnets providing magnetic fields with orthogonal axes; and a housing block, attached to the outer surfaces of the test section, containing 4 Honeywell SS495A Hall-effect sensors in a cross-axial configuration. (**C**) Benchmark stagnant-water rod dynamics: time-series, displacement PDF, PSD, and autocorrelation, confirming stochastic system noise and RMS displacement error < 40 μm. (**D**) Validation setup using synchronized optical and Hall-effect system measurements. (**E**) Consistency in measured vibration dynamics using optical and Hall-effect system (PSD and phase-space attractors) for single-phase water measurements at Reynolds number of 65k (clamped-free rod). (**F**) Complete cross-validation of RMS displacements and frequencies between optical and Hall-effect methods across the whole range of Reynolds number values and flow directions.

We carried out experiments using a custom-built flow system with a vertical test section (Fig. 1A; see Materials and Methods). The test section consists of a cantilevered rod, clamped at the top end and free at the bottom, positioned centrally within a confining tube, hence forming an annular flow passage (see supplementary material, Fig. S1). The system enabled bidirectional flow configurations: for the downward flow (clamped-free) configuration, fluid enters from the top and exits at the bottom; whereas for the upward flow (free-clamped) configuration, the direction is reversed. Axial flow through the annular gap excites rod vibrations via competing mechanisms–stochastic forces (turbulent buffeting, flow unsteadiness, and bubble impacts), movement-induced forces (flutter), and flow separation at the rod tip. To enable two-phase mixtures, air is introduced into the water flow via an upstream mixing section, with the superficial velocities for air ($\:{U}_{gs}$) and water ($\:{U}_{ls}$) being calculated as $\:{U}_{gs}={Q}_{gs}/{S}_{ann}$ and $\:{U}_{ls}={Q}_{ls}/{S}_{ann}$, where $\:{Q}_{gs}$ and $\:{Q}_{ls}$ are the volumetric flow rates of air and water phases, respectively, while $\:{S}_{ann}$ is the cross-sectional area of the annulus flow passage. The homogeneous void fraction (not considering the slip between the phases), $\:\alpha\:$, is thus defined as:

1 $$\:\alpha\:=\frac{{U}_{gs}}{{U}_{gs}+{U}_{ls}}=\frac{{Q}_{gs}}{{Q}_{gs}+{Q}_{ls}}$$

and the annulus Reynolds number, $\:{Re}_{als}$, is defined here using the water phase:

2 $$\:{Re}_{als}=\frac{\rho\:\:{U}_{ls}{\:D}_{hyd}}{\mu\:}$$

where $\:{D}_{hyd}$ is the hydraulic diameter of the annulus, $\:{U}_{ls}$ is the water-phase superficial velocity, while $\:\rho\:$ and $\:\mu\:$ are the density and dynamic viscosity of water identified from measuring its temperature. Experiments span $\:{Re}_{als}$ values of 25k–72k, while covering eight air injection rates, including a single-phase water flow baseline. As such, the homogeneous void fraction ($\:\alpha\:$) has values spanning 0–0.51, dependent on the $\:{Re}_{als}$. Since the rig was operated at ambient temperature, the Reynolds number range covered does not correspond to nuclear reactor operating conditions, where the temperature is higher and the water viscosity is correspondingly smaller. The void fraction range covered, on the other hand, is representative of actual boiling channels. Flow regime maps (see Supplementary Material, Fig. S2), adapted from Hewitt and Roberts^44^ and Taitel^43^, show that higher $\:{Re}_{als}$ cases predominantly fall within the bubbly flow regime, while lower $\:{Re}_{als}$ values approach the slug/churn flow regime, matching with the flow patterns visually observed during our experiments. Finally, regarding resonance, which plays a key role in crossflow FIV^47^, where vortex shedding is the dominant source of excitation, its relevance in axial-FIV is expected to be minor, particularly for the present test setup where the absence of crossflow and the tight space confinement prevent the formation of a coherent vortex street.

To resolve rod vibrations in opaque two-phase flows, we developed a non-intrusive displacement measurement method, leveraging the electromagnetic Hall effect–a well-known phenomenon, where charge carriers in a semiconductor material are deflected by magnetic fields, generating a transverse voltage proportional to the field strength. In our system, displacement is inferred from this voltage output, as magnetic flux density decays with distance from the source. The measuring system consists of a cylindrical mount piece attached to the rod’s free end, embedded with neodymium magnets (model: RS PRO, 4 mm diameter), and a housing block attached to the Perspex test section containing four Hall-effect sensors (model: Honeywell SS495A), Fig. 1B. The sensors detect magnetic field variations induced by rod displacement, enabling high-resolution tracking of the motion of the rod tip. The cylindrical magnet mount and cross-axial sensor alignment ensure uniform sensitivity to bidirectional vibrations. This configuration circumvents bubble-induced optical obstructions, which render optical tracking unfeasible, while maintaining comparable measurement accuracy. In fact, after static calibration the system achieves sub-40 μm measurement precision, as confirmed through repeated experiments in stagnant-water (results exemplified in Fig. 1C). Time-series data, displacement probability density function (PDF), power spectral density (PSD), and autocorrelation functions (ACF) confirm the benchmark noise characteristics: displacements exhibit Gaussian distributions, broadband PSD spectra devoid of dominant frequencies, and rapidly decaying ACF with no periodic coherence–all hallmarks of stochastic system noise.

The Hall-effect system has been validated against synchronized optical tracking in single-phase water flows. For the optical system, a Sony Alpha A6300 camera (100 fps) with LED backlighting and a 45° mirror were used to capture rod motion in orthogonal planes (Fig. 1D). Rod displacements were processed using a custom image-processing algorithm^32,33,48–50^, which extracts the locations of the rod centroid within interrogation windows (Fig. 1D). Simultaneously, Hall-effect measurements have been acquired for direct comparison. Tests were conducted for the conical-tip rod, in both clamped-free and free-clamped configurations, at annulus Reynolds numbers of 25–91k. Results showed strong agreement between the two methods (Fig. 1E,F): radial RMS displacements ($\:R/{D}_{rod}$) showed deviations of less than 5% for Reynolds number above 50k, increasing to 10–20% at lower Reynolds number values due to reduced signal-to-noise ratios. Dominant vibration frequencies matched within 2%, while PSD and phase-space attractors (Fig. 1E) further confirmed dynamic consistency. Note that the present Hall-effect-based sensor was conceived for cantilever rod testing at ambient temperature; extensions to more challenging conditions (multi-rod configurations and/or higher temperature) are clearly feasible, albeit application under realistic nuclear reactor conditions is beyond the scope of the sensor.

As can be noted in Fig. 1E, the PSDs provided by the Hall-effect sensor and by optical imaging are very similar for frequencies below about 10 Hz, indicating that both methodologies successfully capture the dominant vibration frequency of the rod. At higher frequencies, the PSD of the Hall-effect sensor presents a slightly higher energy content with respect to the optical methodology, suggesting that the Hall-effect methodology might be slightly more sensitive to noise than the optical methodology; this part of the spectrum is however of less immediate relevance for the characterization of the rod vibration. Regarding the minor differences in RMS displacements produced by the Hall-effect and optical methodologies that can be noted in Fig. 1F at low Reynolds number values, these are well within the measuring errors.

### Flow-vibration coupling in two-phase flows

**Fig. 2:**
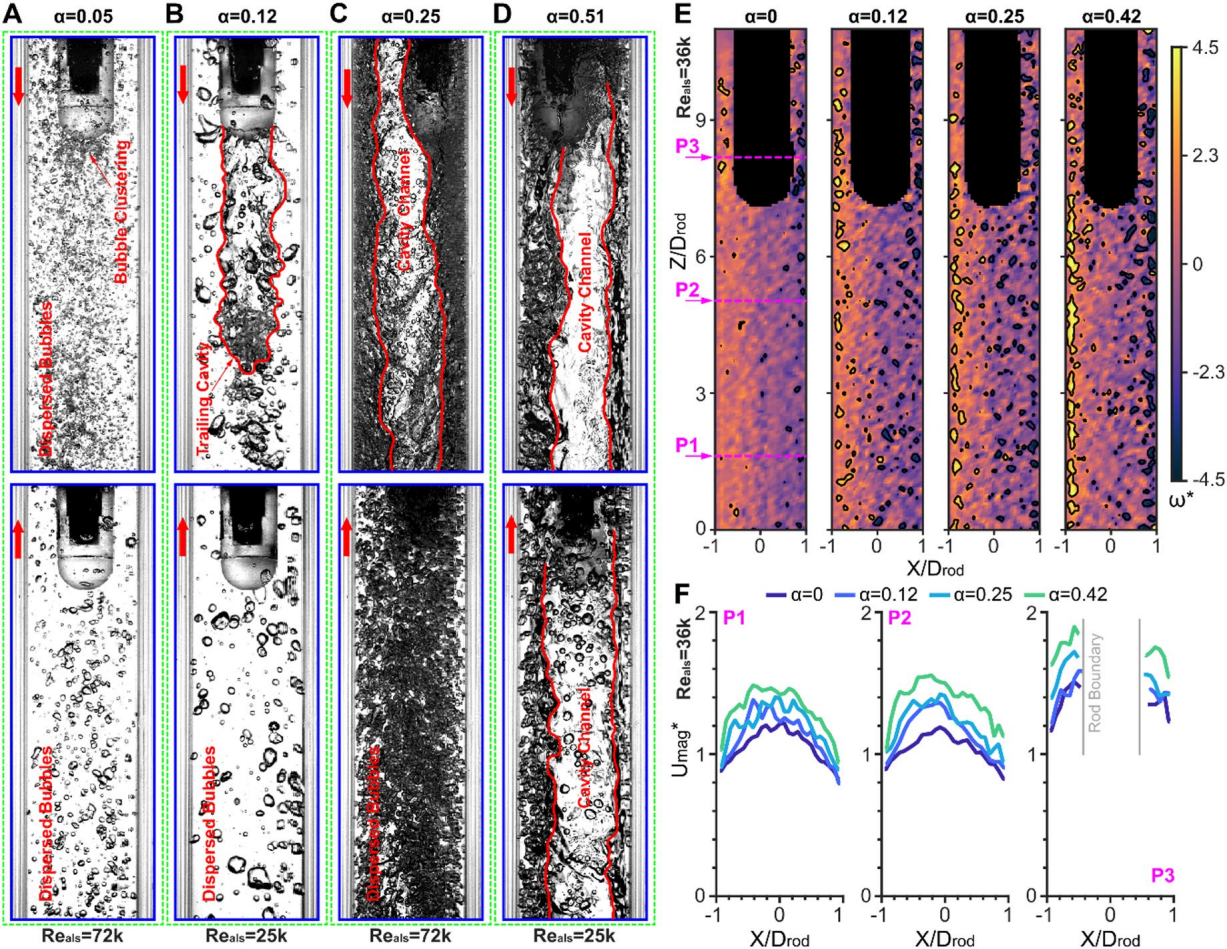
Two-phase flow regimes and velocity field characterization. (**A**–**D**) Flow pattern snapshots (exemplified using the hemispherical rod tip) for increasing homogeneous void fractions $\:{\upalpha\:}$=0.05, 0.12, 0.25, and 0.51 (red arrows: flow direction). Columns (**A**), (**C**): high $\:{Re}_{als}$=72k; Columns (**B**), (**D**): low $\:{Re}_{als}$=25k. High-speed recorded images with Phantom v1610 camera at 6000 fps (30-s videos in Supplementary Movies S1-S8) reveal bubble clustering/coalescence into cavity channels at elevated $\:{\upalpha\:}$, while higher $\:{Re}_{als}$ fragments larger air packets into smaller dispersed bubbles. (**E**) Normalised PIV-LIF vorticity fields ($\:{\omega\:}^{*}=\omega\:\cdot\:{D}_{hyd}/{U}_{ls}$) for $\:{\upalpha\:}$=0 (single phase), 0.12, 0.25, 0.42 (free-clamped examples, $\:{Re}_{als}$=36k), averaged over 9 snapshots (2000 µs intervals); contours indicate vortex boundaries identified using the Q-criterion^51^, highlighting turbulence and flow unsteadiness escalates with rising $\:{\upalpha\:}$. (**F**) Extracted flow velocity profiles ($\:{{U}_{mag}}^{*}={U}_{mag}/{U}_{ls}$) at 3 axially-spaced positions (-6.5$\:{D}_{rod}$, -2.5$\:{D}_{rod}$ and +1$\:{D}_{rod}$ from the rod tip), demonstrating increased stochastic fluctuations of flow velocity as $\:{\upalpha\:}$ increases.

The vibrational dynamics of the cantilevered rod in two-phase flows are governed by the interplay between movement-induced fluid forces and stochastic excitations from turbulence and two-phase interactions. Figure 2 illustrates how increasing the homogeneous void fraction ($\:{\upalpha\:}$) fundamentally alters the flow topology and velocity fields, thereby reshaping the flow excitation mechanisms.

At low void fraction ($\:{\upalpha\:}$=0.05; Fig. 2A), in both flow directions, finely dispersed bubbles minimally perturb the flow. While these dispersed bubbles introduce density perturbations, they preserve coherent flow structures and velocity profiles, resulting in pressure/shear stress variations over the rod’s surface similar to those of single-phase conditions, albeit with additional stochastic fluctuations emerging from localised bubble impacts. Particularly, in the clamped-free (down flow) configuration, reduced velocity in the tip wake region promotes transient bubble clustering (Supplementary Movie S1). The bubbles remain dispersed due to low $\:{\upalpha\:}$ and high kinetic energy at the Reynolds number ($\:{Re}_{als}$=72k). At $\:{\upalpha\:}$=0.12, similar dispersed bubbly flows persist in the free-clamped (up flow) configuration, but the clamped-free case exhibits a trailing cavity channel formed by bubble coalescence in the bluff-body wake (Fig. 2B). Notably, even at low $\:{\upalpha\:}$, intermittent large annular air packets traverse the flow passage (Supplementary Movies S1–S4), introducing stochastic vibration excitations.

As the void fraction increases to $\:{\upalpha\:}$=0.25 and 0.51, bubble coalescence dominates the flow, forming cavity channels that occupy large portions of the flow passage (Fig. 2C,D) (however, intermittent dispersed bubble phases are preserved, see Supplementary Movies S5-S8). At lower $\:{Re}_{als}$= 25k (Fig. 2D), inertial forces fail to fragment the large air packets, resulting in persistent cavity channels that disrupt flow coherence. Conversely, at higher $\:{Re}_{als}$= 72k (Fig. 2C), turbulent kinetic energy fragments cavities into smaller, dispersed phases. This regime-dependent behaviour reduces periodic fluid forces due to nonuniform fluid density, while stochastic forces intensify from chaotic inter-phase interactions.

The effects of change in flow regime on flow excitations are further illustrated in Fig. 2E,F, using vorticity/velocity profiles obtained from particle image velocimetry with laser-induced fluorescence (PIV-LIF). Rising $\:{\upalpha\:}$ amplifies vorticity fluctuations (Fig. 2E), while the velocity profiles at three axial locations reveal a simultaneous increase in both the cross-sectionally averaged velocity and velocity fluctuations at higher $\:{\upalpha\:}$ (Fig. 2F). The increased average velocity is a result of reduced mixture density at higher void fractions, whereas the enhanced fluctuations in both vorticity and velocity profiles originate from the presence of larger bubbles, which intensify the mixing of momentum and disrupts the effects of viscous forces. As a result, stochastic excitations increase with rising $\:{\upalpha\:}$. The combined effects of two-phase flow led to the dual-effect on FIVs–stochastic excitations were enhanced by turbulence amplification and bubble impacts, while movement-induced forces attenuated due to density nonuniformities.

**Fig. 3:**
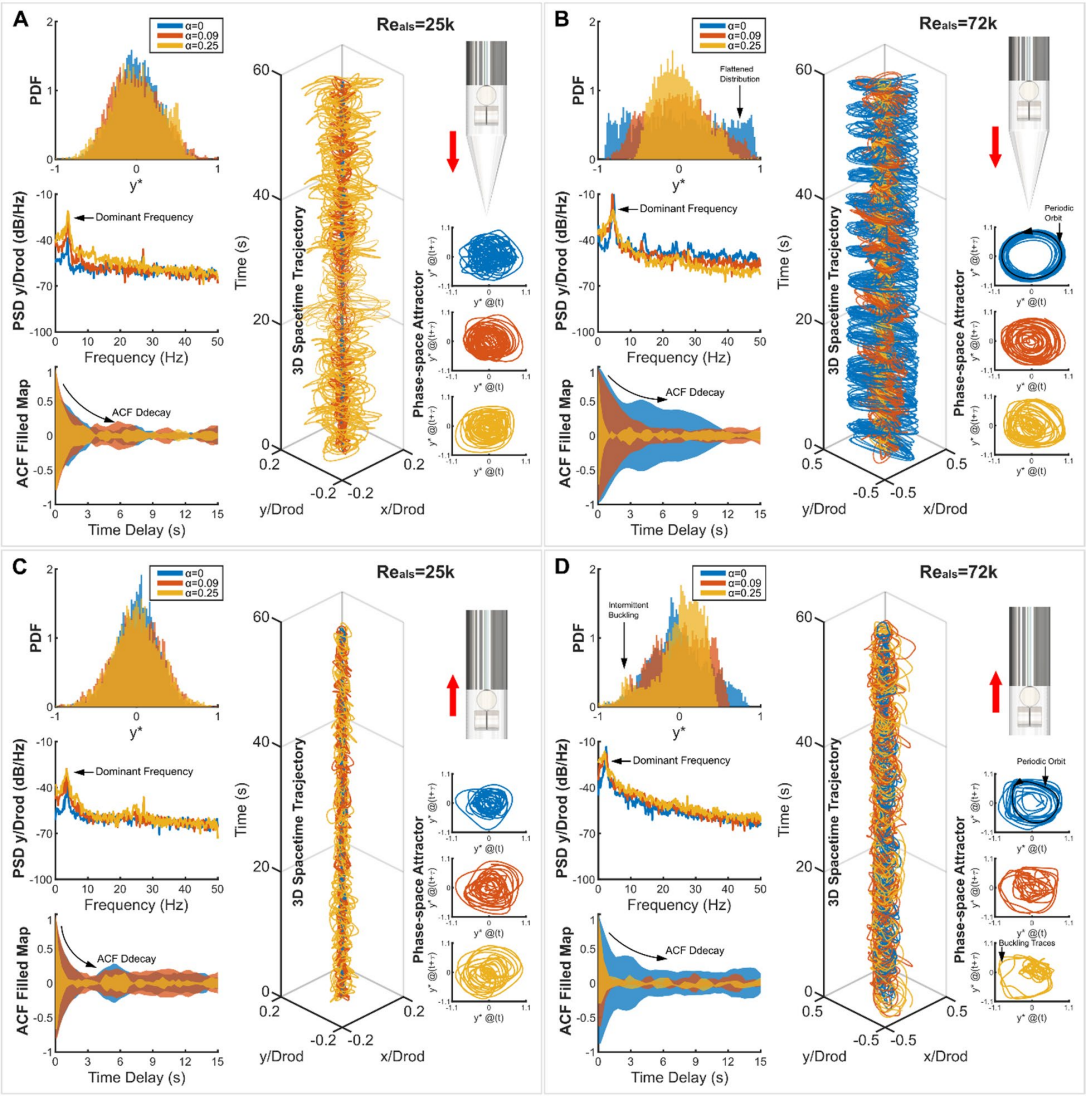
Competing stochastic and periodic rod vibration dynamics across two-phase flow regimes and flow/geometry configurations. (**A**, **B**) Clamped-free conical-tip dynamics. Large-amplitude oscillation (flutter) occurs for single-phase flow ($\:{\upalpha\:}$=0) at $\:{Re}_{als}$=72k: flattened PDF, ring-shaped attractor indicating periodic motion, slow-decaying ACF. (**C**, **D**) Free-clamped blunt-tip dynamics. Intermittent buckling^32^ emerges at high $\:{\upalpha\:}$=0.25 and $\:{Re}_{als}$=72k: localised PDF peak region; attractor show traces away from centroid. Key observations: (i) Single-peaked PSD and amorphous attractor topology, signature of fuzzy-period 1 dynamics, except single-phase flutter divergence at $\:{Re}_{als}$=72k (clamped-free conical tip). (ii) Higher $\:{Re}_{als}$, i.e., (**B**), (**D**), leads to enhanced periodic vibrations (flattened/off-centred PDF distribution, slower ACF decay at low $\:{\upalpha\:}$). (iii) Elevated $\:{\upalpha\:}$ universally reduces periodicity and shifts dynamics to more stochastic regimes (Flattened converted to Gaussian PDF profile, more rapid ACF decay, wider PSD peak energy). Visualization details: coordinates normalised for PDF and phase-space attractor ($\:{y}^{*}=y/\left|{y}_{max}\right|$); ACF filled map ignores small-cycle information but emphasises peak/trough decay.

Figure 3 disentangles how these competing excitations govern rod dynamics, exemplified using the clamped-free conical tip and free-clamped blunt tip configurations (see Supplementary Material, Figs. S3, S4 for additional boundary configurations and hemispherical tip data).

In the clamped-free case, single-phase flow at low $\:{Re}_{als}$=25k excites fuzzy period-1 motion^52^, characterised by Gaussian displacement distribution, single-peaked power spectral distribution, and amorphous phase-space attractor (Fig. 3A), driven by stochastic turbulence and weak movement-induced periodic forces^42,53^. Introducing two-phase flows amplifies vibrations via increased stochastic excitations–increased flow turbulence (Fig. 2E) along with stochastic air packet/cavity impacts, while suppressing movement-induced-forcing through phase density contrasts. Consequently, increasing the void fraction, $\:{\upalpha\:}$, elevates vibration amplitudes but decreases periodicity, evidenced by faster autocorrelation decay at $\:{\upalpha\:}$=0.09 and 0.25 (Fig. 3A). At the high $\:{Re}_{als}$=72k, single-phase flow triggers large-amplitude flutter oscillation, marked by flattened PDF distribution, ring-like phase-space attractor, and slow-decaying autocorrelation (Fig. 3B), driven by dominant motion-induced periodic forces. Two-phase flows disrupt this mechanism: stochastic air packets/cavities breakdown coherent flow structures during rod displacements, desynchronise fluid force-rod displacement response while promoting turbulence, thereby reducing vibration amplitude, and reverting dynamics to stochastic dominance. The free-clamped configuration further highlights the two-phase effects: rods generally exhibit fuzzy-periodic dynamics across $\:{Re}_{als}$ (Fig. 3C,D), increasing $\:{\upalpha\:}$ reduces vibrational periodicity (faster autocorrelation decay) while amplifying amplitudes across $\:{Re}_{als}$ through stochastic-forcing.

Rod vibration dynamics exhibits geometry-mediated sensitivity^33^, modulated by flow direction and two-phase flow effects. For clamped-free conical and hemispherical tips (Supplementary Material Fig. S4), the tapered geometry profiles suppress flow separation, increasing periodic-forcing in single-phase flows. However, two-phase flows reduce this effect where gas-liquid interactions overwhelm tip-driven coherent flows, yielding stochastic motions indistinguishable between different tip geometries at high $\:{\upalpha\:}$. Conversely, in free-clamped configurations, stochastic instabilities and unsteady flow separation dominate rod vibrations^33^. The tapered geometry profiles for the conical and hemispherical tips reduce flow excitations; however, two-phase excitations increase across tip geometries, enhancing stochastic vibrations as $\:{\upalpha\:}$ increases. Notably, at $\:{\upalpha\:}$=0.25, autocorrelation decays rapidly in both clamped-free and free-clamped configurations (Fig. 3A–D, Supplementary Material, Figs. S3, S4), signalling a two-phase dominant regime where stochastic excitations override Reynolds number and tip geometry effects.

### General trends of rod vibrations in two-phase flows

**Fig. 4:**
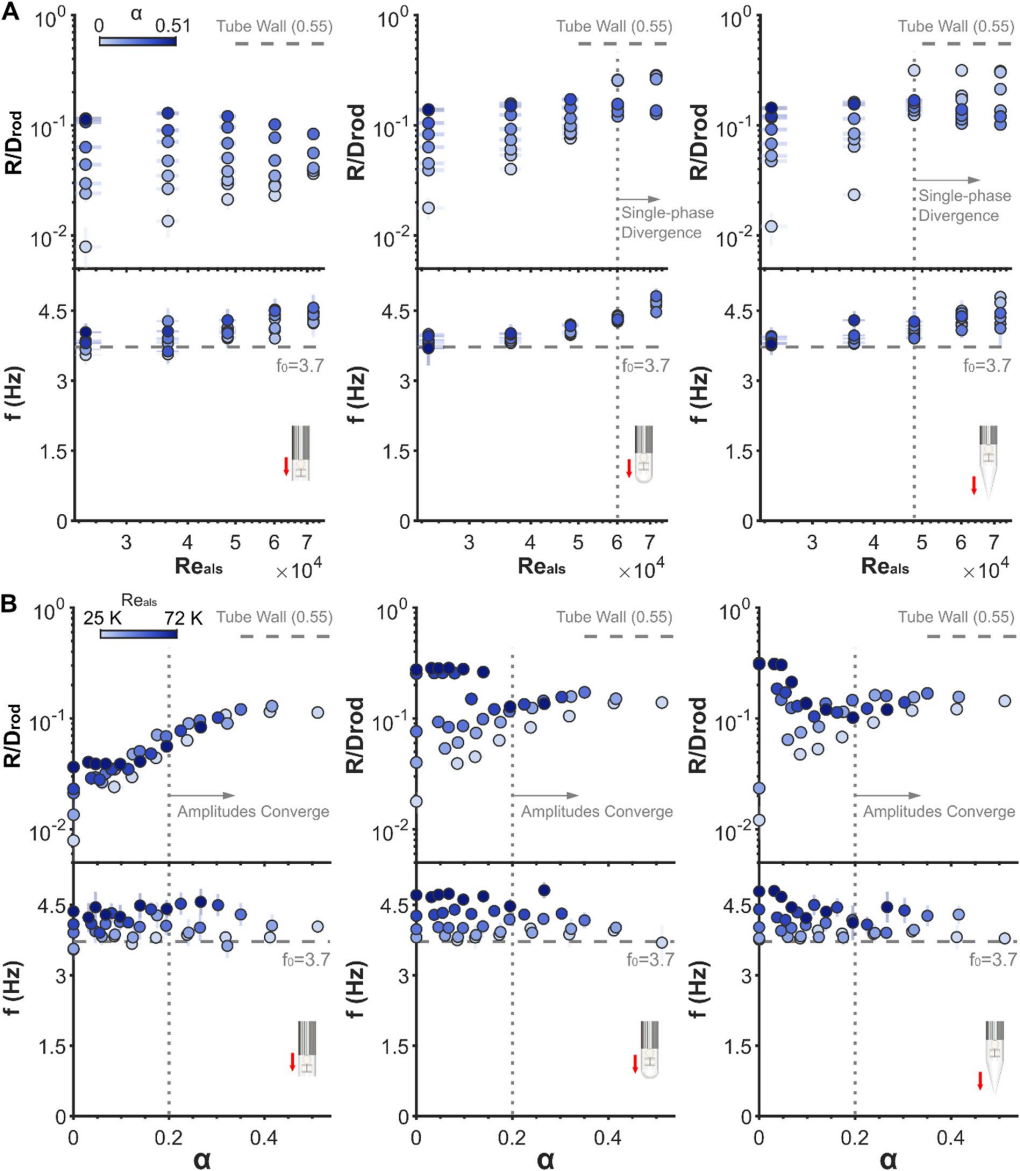
Clamped-free rod amplitude-frequency: Reynolds number and void fraction dependence. (**A**) RMS vibration amplitude and dominant frequency vs. $\:{Re}_{als}$ for $\:\alpha\:$=0–0.51. Single-phase flows ($\:\alpha\:$=0) display linear amplitude growth with $\:{Re}_{als}$, culminating in flutter divergence for hemispherical ($\:{Re}_{als}$=60k) and conical ($\:{Re}_{als}$=48k) tips. Two-phase flows ($\:\alpha\:$>0) suppress this instability, yielding amplitudes plateau/decline despite rising $\:{Re}_{als}$. (**B**) Amplitude–frequency vs. $\:\alpha\:$ at $\:{Re}_{als}$ = 25k–72k. Amplitudes show $\:{Re}_{als}$-dependent trends for $\:\alpha\:$<0.2, increasing at low $\:{Re}_{als}$ (stochastic forcing dominance) but saturating/decreasing at high $\:{Re}_{als}$ (periodic forcing suppression); above $\:\alpha\:$=0.2, amplitude values converge across tip geometries and $\:{Re}_{als}$.

**Fig. 5:**
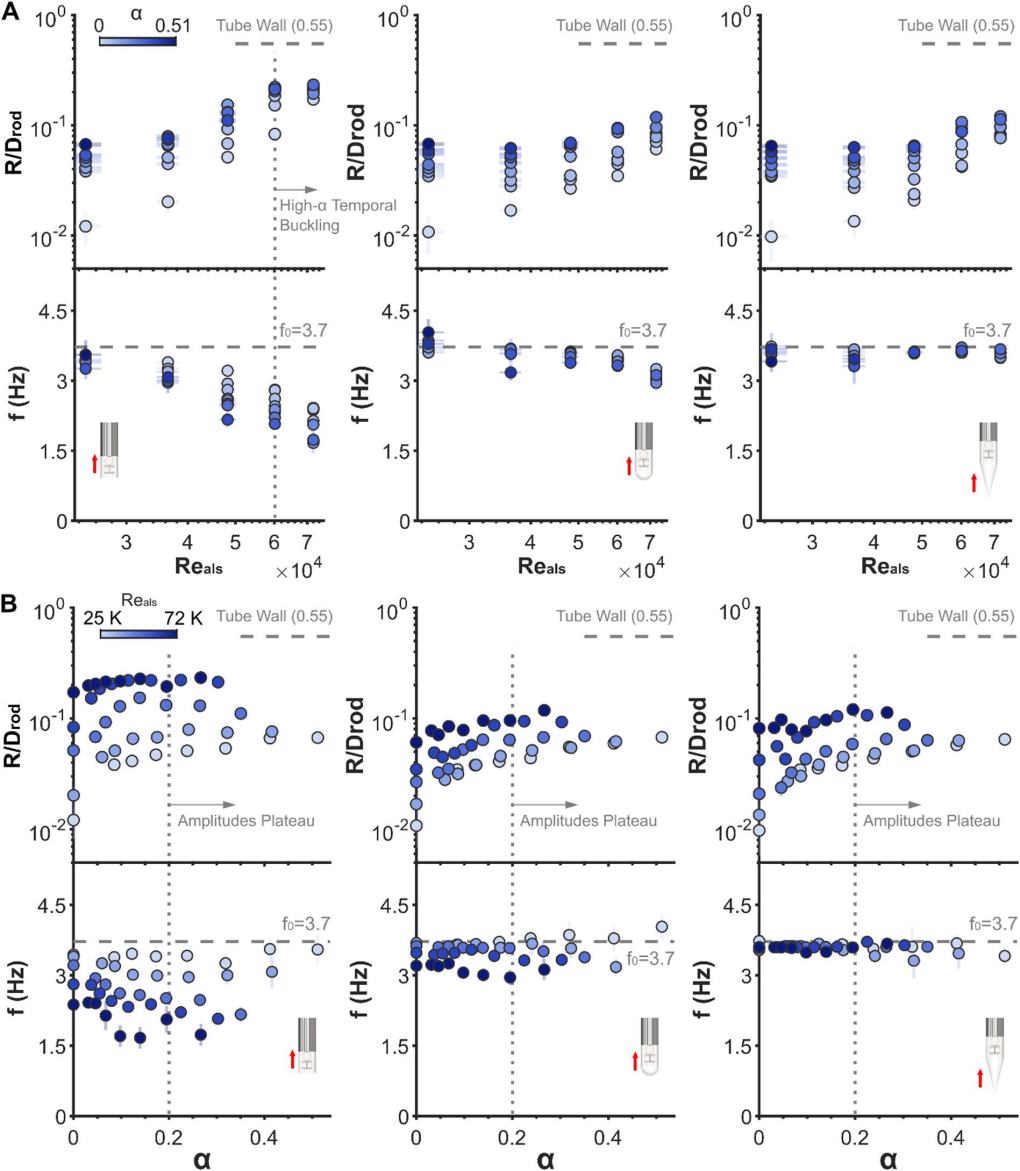
Free-clamped rod amplitude-frequency: Stochastic universality. (**A**) RMS amplitude and dominant frequency vs. $\:{Re}_{als}$ for $\:\alpha\:$=0–0.51. Amplitude growth weakens with rising $\:\alpha\:$ as two-phase driven stochastic effects reduce $\:{Re}_{als}$ dependence. Intermittent buckling induced by two-phase flows ($\:\alpha\:$>0.2) for blunt tip at $\:{Re}_{als}$=60k due to two-phase stochastic amplification. Frequencies decline with $\:{Re}_{als}$, contrasting clamped-free rods. Tapering of tip geometry leads to less frequency reduction (e.g., conical tips). (**B**) Amplitude-frequency scaling with $\:\alpha\:$ at $\:{Re}_{als}$=25k–72k. Amplitudes increase gradually for $\:\alpha\:$<0.2 but saturate for $\:\alpha\:$>0.2 at high $\:{Re}_{als}$, consistent with clamped-free rods, signalling universal stochastic dominance regime above $\:\alpha\:$=0.2.

This section synthesizes the global vibration response of cantilever rods in two-phase axial flows, revealing the competing effects between periodic movement-induced forcing and stochastic excitations for FIV dynamics in air-water flows. Figures 4 and 5 map this interplay across Reynolds numbers ($\:{Re}_{als}$), homogeneous void fractions ($\:\alpha\:$), tip geometries, and flow configurations.

In single-phase flows, rods subjected to clamped-free axial flows exhibit distinct tip geometry dependent oscillatory behaviours. Blunt and hemispherical tips show linear amplitude growth with $\:{Re}_{als}$ (log-log scale, Fig. 4A), with higher amplitudes for the hemispherical tip, culminating in flutter divergence at $\:{Re}_{als}$=60k. In contrast, the conical tip undergoes a sudden instability jump at $\:{Re}_{als}$=48k, beyond which flutter divergence occurs. Two-phase flows ($\:\alpha\:$>0) homogenise these tip geometry effects: amplitude growth with $\:{Re}_{als}$ diminishes as $\:\alpha\:$ increases, and flutter instability, driven by periodic fluid forcing^32,33^, vanishes in two-phase flows. Instead, amplitude plateaus (or decreases at high $\:\alpha\:$) with rising $\:{Re}_{als}$, demonstrating gas-liquid interactions suppresses periodic excitations. Conversely, in free-clamped configurations (Fig. 5A), single-phase amplitudes rise monotonically with $\:{Re}_{als}$ and consistently across different tip shapes. Two-phase flows ($\:\alpha\:$>0) amplify amplitudes across all tip geometries and $\:{Re}_{als}$, inducing intermittent buckling for blunt tip rods at $\:\alpha\:$>0.2 and $\:{Re}_{als}$>60k, highlighting enhanced stochastic excitations via gas-liquid interactions. Notably, the absence of instabilities (periodic flutter or divergence) for the blunt tip rods in the clamped-free configuration generalizes theoretical predictions and experimental observations so far restricted to single-phase flows^52^.

Amplitude scaling with the void fraction, $\:\alpha\:$, reveals strong dependencies on tip geometry and $\:{Re}_{als}$. For clamped-free blunt tips (Fig. 4B), amplitudes generally increase with $\:\alpha\:$ at all Reynolds numbers, $\:{Re}_{als}$, whereas hemispherical and conical tips exhibit amplitude growth at low $\:{Re}_{als}$ but reductions at high $\:{Re}_{als}$. This reflects a reduction in periodic excitation and enhancement in stochastic excitations as $\:\alpha\:$ increases. Strikingly, clamped-free amplitudes converge across tip geometries and $\:{Re}_{als}$ for $\:\alpha\:$>0.2, signalling two-phase driven universal stochastic dominance regime. Free-clamped amplitude scaling diverges from this trend (Fig. 5B): amplitudes generally increase gradually with $\:\alpha\:$ for $\:\alpha\:$<0.2 (independent of tip geometry and $\:{Re}_{als}$) but plateau at elevated $\:{Re}_{als}$ for $\:\alpha\:$>0.2. While convergence in amplitudes emerges, it progresses with $\:\alpha\:$ at a slower rate. The observed discrepancy originates from distinct single-phase excitation mechanisms at elevated $\:{Re}_{als}$: clamped-free rods exhibit periodic dominance through motion-induced forces, while free-clamped rods are more influenced by stochastic effects via turbulence and unsteady flow separation. This dichotomy persists in two-phase flows where periodic dominance rapidly decays as two-phase driven stochastic effects intensify, whereas stochastic turbulence and unsteady effects remains resilient to two-phase perturbations.

Vibration frequencies for both configurations remain anchored near the rod’s fundamental natural frequency ($\:{f}_{0}$=3.7 Hz). When the flow direction is from the clamped to the free end of the rod, the frequency gradually increases with $\:{Re}_{als}$ across tip geometries, whereas when the flow direction is reversed, the frequency decays with $\:{Re}_{als}$. These phenomena are analogous to the behaviour of a column under tension or compression: tension increases effective stiffness (and thus natural frequency), while compression reduces them. A classic example is the centrifugal stiffening of helicopter blades, where centrifugal forces impose tensile loads that resist bending and increase blade natural frequencies. Specifically, fluid forces acting on clamped-free rods resist displacement, effectively increasing stiffness and frequencies, whereas fluid forces on free-clamped rods facilitate displacement, thereby enhancing damping and reducing frequencies. The confinement effects of the outer tube are also likely to contribute to these trends. Such damping effects diminish with increased taperness of tip geometry, leading to nearly frequency invariance for conical tips. Notably, rod vibration frequencies generally show minimum dependence on $\:\alpha\:$, with only random deviations observed, further underscoring the stochastic nature of two-phase excitations.

Collectively, these results demonstrate that two-phase flows impose dual competing effects on axial-FIVs: (i) stochastic excitations escalate with $\:{\upalpha\:}$ due to gas cavity impacts and two-phase driven turbulence, amplifying vibration amplitudes at low $\:{Re}_{als}$, and (ii) motion-induced periodic forces diminish as two-phase flows disrupt coherent structures, suppressing flutter divergence at high $\:{Re}_{als}$. This trade-off defines a critical threshold of $\:{\upalpha\:}$=0.2, beyond which stochastic effects dominate, overriding flow configuration, Reynolds number, and tip geometry–a shift from single-phase FIV case, where periodic instabilities govern large amplitude vibrations and consequently failure mechanisms.

By offering insight into the fundamental physics of two-phase axial-FIVs, the present findings are of relevance for the nuclear and marine industries where two-phase flows are commonplace, despite the simple paradigmatic experimental setup employed. The dominant stochastic regime above $\:{\upalpha\:}$=0.2 indicates that design optimizations effective with single-phase flows, e.g., tip geometry refinement, become less effective in high-$\:{\upalpha\:}$ environments, necessitating new strategies, e.g., turbulence baffling/damping and/or gas cavity fragmentation. The dual role of two-phase flows–amplifying stochastic vibrations while suppressing periodic instabilities–highlights the need for condition-specific strategies to manage flow-induced vibrations in two-phase flow environments. The results also provide useful datasets for development and validation of numerical simulation methods.

Future investigations should include: (i) multi-rod assembly dynamics, where shadowing effects, wake-driven instabilities, and bundle synchronization dictate system-level vibrational cascades; (ii) embedded sensing networks, while adapting the proposed Hall-effect sensing approach into wireless, miniaturized sensors for real-time FIV monitoring in more complex environments of more immediate industrial relevance; (iii) varying the mass inside the rod (by adding/removing lead shot) to investigate the effect of mass/inertia; and (iv) investigating gravity effects at various Reynolds numbers, e.g., through flipping the clamping position to be at the bottom of the tube.

Another direction of future work is to further consider the effect of non-dimensional parameters relevant to the current problem. In addition to the Reynolds number, we have previously demonstrated the importance of another dimensionless parameter that involves the fluid velocity and the component modulus of elasticity, i.e., the Cauchy number (representing the dynamic pressure to solid elasticity ratio)^39^. Experimental investigations, including ours, have so far been unable to separate the effects of this dimensionless number from those of the flow Reynolds number. As such, as an important direction for future work, experimental rig capabilities should be extended to enable the control of the working fluid temperature, hence the Reynolds number, while using the velocity to control the Cauchy number. This way, the Reynolds and Cauchy numbers, which both include velocity, will be varied independently, which is expected to be critical to the development of scaling laws. In the case of gas-liquid two-phase flows, clearly an additional dimensionless number would be the void fraction.

## Materials and methods

### Experimental rig

The flow system, driven by a centrifugal pump (10HP Leeson), builds on a prior configuration custom-built for axial FIV experiments on cantilever rods with single-phase water flow^32,33^. The rig was upgraded by incorporating flow paths for controlled air injection (Fig. 1A) thereby enabling two-phase air-water flow experiments. Water temperature was monitored using a digital immersion thermometer (ETI Ltd; 248–343 K range, ± 1 K accuracy), which informed real-time estimates of fluid density and viscosity. A turbine flow meter (Cole-Parmer; 37.8–378 L/min range, ± 3.78 L/min accuracy) positioned upstream of the test section measured water flow rates. Compressed air (4 Bar supply) was regulated via a SMC PFM750S flow meter (0–50 L/min range, ± 0.5 L/min). A circumferential injection manifold featuring four valved ports was used to achieve uniform air-water phase distribution (Fig. 1A).

The test section included a rigidly mounted stainless-steel confining tube (AISI 316; 20.8/25 mm inner/outer diameter, Supplementary Material Fig. S1A). For optical access, a transparent Perspex segment, matching the confining tube in diameter, replaced the steel tube near the rod tip, and was surrounded by a water-filled square Perspex box to eliminate cylindrical lensing and image distortion (Supplementary Material Fig. S1A). Flow conditioning was achieved via a tube-bundle flow straightener comprising three internal pipes (100 mm length) installed at the test section base to suppress swirl and secondary flows. The free-clamped configuration maintained an upstream recovery length of 1090 mm (> 50 pipe diameters) which further ensured the establishment of fully developed single-phase flows, whose attainment was demonstrated in prior studies^32,33^. The clamped-free configuration omitted the flow straightener, as the flow originated from two symmetric streams merging at the union cross (Swagelok SS-25M0-4), with upstream perturbations exerting minimal influence.

### Test rod

The test rod was made from AISI 316 stainless-steel tubing (8.8/10 mm inner/outer diameter) with a density of 7.99 g/cm^3^ and a Young’s modulus of 193 GPa. The rod was internally packed with lead shot (0.3–1.6 mm diameter, 11.34 g/cm³ density) to simulate nuclear fuel pellet loading. Homogeneity tests confirmed a centre-of-mass shift < 0.5% from the centre. Lead shot packing (84.6% ratio) ensured static internal loading without altering flexural rigidity, as verified through free-vibration experiments^32^. The measured linear mass density of the rod, accounting for both the rod material and the lead shot filling, is 5.88 g/cm. This compares well to actual fuel reactor rods within PWR which typically have diameters between 9 and 11 mm and a linear mass density from 5 to 7 g/cm. Notably, the rod vibration was first assessed using free vibration tests in stagnant water, where the rod was manually displaced from its resting position. Recording and analyzing the resulting free vibration, the measured natural frequency was found to be 3.7 Hz and the damping ratio was found to have a small value of 0.012. Both experimentally identified quantities were in strong agreement with theoretical models^32^.

Interchangeable rod tips (conical, hemispherical) were mounted on a blunt mount-piece (Fig. 1B), which was affixed to a lightweight aluminium cap (2.1 g) used to seal the rod (Supplementary Material Fig. S1B). Both the mount-piece and the rod-tips were 3D-printed, using light-weight hard resin with negligible mass, to ensure minimal influence on vibration dynamics. The test rod was placed inside the test tube as a vertical cantilever beam fixed at the top and free at the bottom, with air-water flowing in the annular gap between rod and tube. The hydraulic diameter of the annular gap was 10.85 mm, a value representative of water-cooled nuclear reactor cores. The test piece was carefully designed to yield a paradigmatic test configuration whose geometry, structural properties, and fluid excitation mechanisms (turbulent buffeting, motion-induced excitation, and unsteady flow separation) are as informative as feasible of actual nuclear reactor cores. Detailed dimensions of the experimental components are provided in Supplementary Material Fig. S1.

### Vibration measurement protocol

Rod vibration displacements were tracked using a custom-built Hall-effect based toolkit (Fig. 1B), comprising four Hall-effect sensors interfaced with a National Instruments USB-6210 data acquisition system (1 kHz sampling rate). Pre-experiment calibration mapped 120 gridded positions in a planar Cartesian coordinate system (Supplementary Material Fig. S5), spanning ± 7 mm lateral distance to the confining tube centreline–a range exceeding the rod’s maximum allowable displacement (± 5.5 mm) within the tube. Each location was sampled over 60-second intervals (60,000 data points per sensor) to generate averaged reference values. This calibration matrix enabled precise spatial mapping between sensor outputs and rod tip coordinates via biharmonic spline interpolation.

Raw displacement time series were filtered using a sixth-order Butterworth low-pass filter (50 Hz cutoff), eliminating noise components well above the system’s fundamental natural frequency (3.7 Hz). Filtered data were transformed into physical coordinates using the calibration matrix. The trajectories were analysed via Welch’s method^54^ for power spectral density (PSD) estimation, autocorrelation functions (ATC) to assess temporal coherence, and phase-space attractor reconstruction to characterize dynamical behaviour^55^. Frequency uncertainties were quantified as half of the full-width-at-half-maximum (FWHM) of the spectral peaks; RMS amplitudes were derived directly from the time series. Static controlled experiments (stagnant water, pump off) confirmed a baseline RMS displacement error < 40 μm. For completeness, Table 1 shows the absolute and derived uncertainties involved.

**Table 1:** Absolute and derived uncertainties (employing standard error propagating techniques).

Absolute uncertainties		
Quantity	Symbol	Value
Water flow rate	$\:{\delta\:Q}_{ls}$	± 3.78 L/min
Air flow rate	$\:{\delta\:Q}_{gs}$	± 0.5 L/min
RMS rod displacement	$\:\delta\:\mathrm{R}$	0.04 mm (via still water tests)

Derived parameter errors			
Quantity	Symbol	Value	Formula used
Superficial water velocity	$\:{\delta\:U}_{ls}$	± 0.1846 m/s	$\:{\delta\:U}_{ls}={\delta\:Q}_{ls}/{S}_{ann}$
Superficial air velocity	$\:{\delta\:U}_{gs}$	± 0.0244 m/s	$\:{\delta\:U}_{gs}={\delta\:Q}_{gs}/{S}_{ann}$
Reynolds number	$\:\delta\:\mathrm{R}\mathrm{e}$	Approx. ±2700	$\:\delta\:\mathrm{R}\mathrm{e}=\mathrm{R}\mathrm{e}\:({\delta\:U}_{ls}/{U}_{ls})$
Void fraction	$\:\delta\:{\upalpha\:}$	± 0.0000-0.0131	$\:\delta\:{\upalpha\:}={\upalpha\:}({\delta\:U}_{gs}/{U}_{gs})$
Frequency	$\:\delta\:f$	± 0.1031–0.7640 Hz	half of Full Width at Half Maximum (FWHM)

### Flow field measurement

Air-water two-phase flow velocity fields were measured using particle image velocimetry combined with laser-induced fluorescence. Fluorescent PMMA-RhB-FRAK particles (1–20 μm diameter, DANTEC DYNAMICS; 25% w/v suspension; abs/em 560/584 nm) were excited by a 527 nm Nd: YLF laser (15 mJ/pulse, 30 µs pulse delay). A two-dimensional laser sheet (1 mm thickness) illuminated the flow field near the rod tip, with a mirror positioned at the obscured-side to enhance visibility (Supplementary Material Fig. S6). Particle dispersion was optimized by operating the rig for several minutes at maximum flow rate prior to experiments. A high-speed camera (Phantom V310; 1280 × 800 pixels, Nikon AF Micro 60 mm f/2.8D lens) equipped with a 560 nm long-pass filter, captured seeded flow images, with scattered light at the laser wavelength filtered out and only the light fluoresced by the seeding particles remained.

Image processing utilized the PIVLab toolbox^56^: raw images were high-pass filtered (5-pixel kernel) to enhance particle contrast. Interrogation windows (32 × 32 pixels, 50% overlap) and FFT cross-correlation generated velocity vector fields, with spurious vectors (correlation coefficient < 0.4) removed. Nine instantaneous measurements with 2000 µs intervals (during which the flow traversed significant distances while the rod remained nearly stationary) were averaged per case to generate smooth-instantaneous velocity fields, mitigating bubble-induced irregularities.

It is worth mentioning that optical obstruction caused by bubbles presents a significant measurement challenge, particularly at the higher void fractions (e.g., alpha > 0.25). In these conditions, bubbles not only block the laser sheet but also generate noisy scattered light, rendering standard PIV algorithms ineffective. As such, our results focus on qualitative diagnostics rather than quantitative analysis. The primarily strategies we employed to mitigate optical interference included both spectral and temporal filtering. For spectral filtering, as mentioned previously, we used laser-induced fluorescence (LIF) with Fluorescent PMMA-RhB-FRAK particles of emission wavelength 560–584 nm, excited by a 1 mm Nd: YLF laser sheet of wavelength 527 nm. We also equipped the camera with a long-pass filter, which successfully blocked light with wavelengths below 560 nm. This effectively removed most of the noisy light scattered by the bubbles (whose wavelength remains near 527 nm) while retaining the signal from the fluorescent tracer particles. For temporal filtering, we implemented a carefully designed time-window for frame averaging. We captured nine snapshots at 2000 µs intervals. Due to the large ratio between the flow frequency/velocity and the vibrating rod (approx. 3.7 Hz, near the fundamental natural frequency), the rod remained effectively stationary during the short capture window, while the flow traversed significant distances. This allowed us to perform field averaging where the rod’s position was nearly fixed, but the bubbles had moved, effectively averaging out ‘blackout areas’ caused by large bubbles. As such, the vorticity field was derived from these averaged results in the same manner as the single-phase case.

## Supplementary Information

Below is the link to the electronic supplementary material.

Supplementary Material 1

Supplementary Material 2

## Data Availability

All data are available in the main text or the supplementary materials.
