# Supplementary material for: Unveiling the fundamentals of two-phase axial-flow-induced vibrations of cantilever rods
Source: Sci Rep. 2026 Jan 13;16:5102. doi: 10.1038/s41598-026-35337-4 (PMC12876934; doi:10.1038/s41598-026-35337-4)
Supplement: Supplementary file 1 — Supplementary Material 1 [file 41598_2026_35337_MOESM1_ESM.pdf]

Supplementary Materials for

Unveiling the fundamentals of two-phase axial-flow-induced vibrations of cantilever rods

Hao Li[1,2], Andrea Cioncolini[3], Hector Iacovides[1], William Benguigui[4,5], Mostafa R.A. Nabawy[1,6]*

[1] Department of Mechanical and Aerospace Engineering, University of Manchester, Oxford Road, M13 9PL Manchester, UK.
[2] School of Transportation Science and Engineering, Beihang University, 100191 Beijing, China.
[3] Department of Mechanical Engineering (Robotics), Guangdong Technion - Israel Institute of Technology (GTIIT), 241 Daxue Road, 515063 Shantou, Guangdong, China.
[4] EDF R&D, Fluid Mechanic division, 6 Quai Watier 78400 Chatou, France.
[5] IMSIA, UMR EDF/CNRS/ENSTA 9219, Université Paris-Saclay, Palaiseau, France.
[6] Aerospace Engineering Department, Faculty of Engineering, Cairo University, Giza 12613, Egypt.

*Corresponding author: Mostafa R.A. Nabawy. Email: mostafa.ahmednabawy@manchester.ac.uk.

This PDF file includes:

Figs. S1 to S6
Legends for movies S1 to S8
Legend for data S1

Other Supplementary Materials for this manuscript include the following:

Movies S1 to S8
Data S1

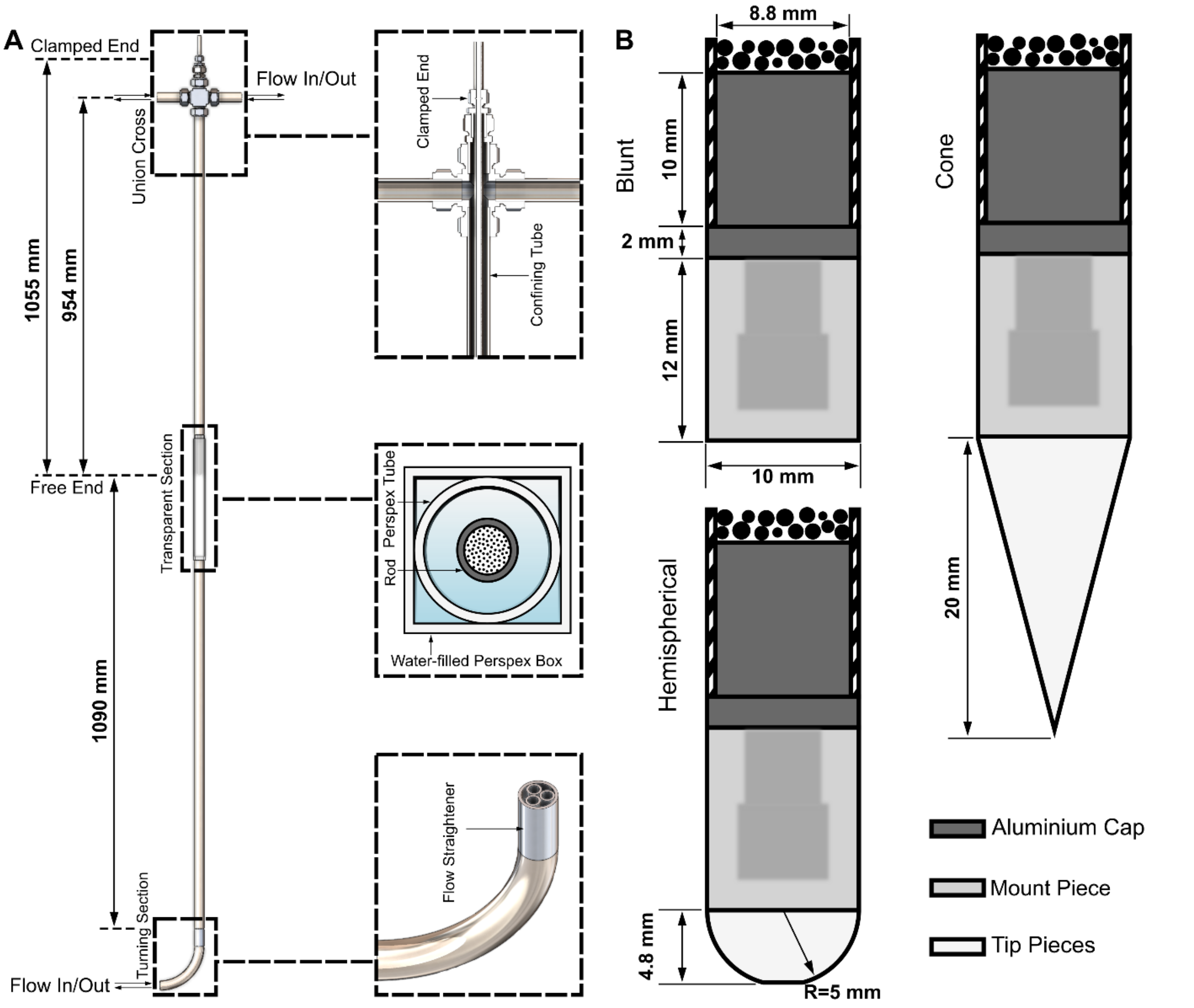

Fig. S1. Schematics of the experimental test section and rod tip configurations. (**A**) Diagram detailing the components of the test section, highlighting the rod/tube lengths specific to the blunt-tip setup (rod length includes mount piece); (**B**) Illustrations and dimensions of different rod tip designs, including blunt, hemispherical, and conical tips.

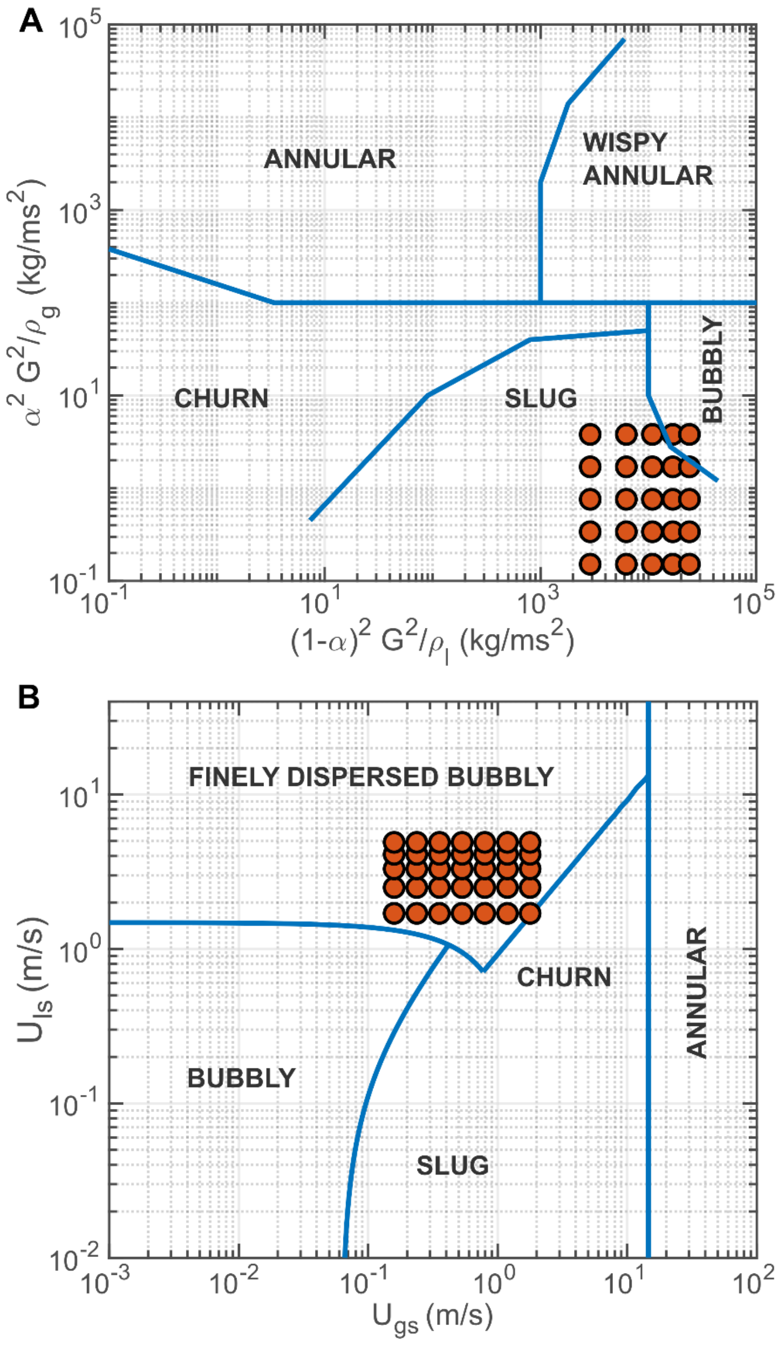

Fig. S2. Experimental two-phase flows mapped onto established flow regime diagrams. Experimental cases overlaid on: (**A**) the Hewitt and Hall-Taylor *(44)* flow regime map and (**B**) the Taitel et al. *(43)* flow regime map. Note that for consistency with the literature, superficial velocities are defined based on the tube area ($\boldsymbol{U_{gs} = Q_{gs}/S_{tube}}$; $\boldsymbol{U_{ls} = Q_{ls}/S_{tube}}$), rather than the annulus area.

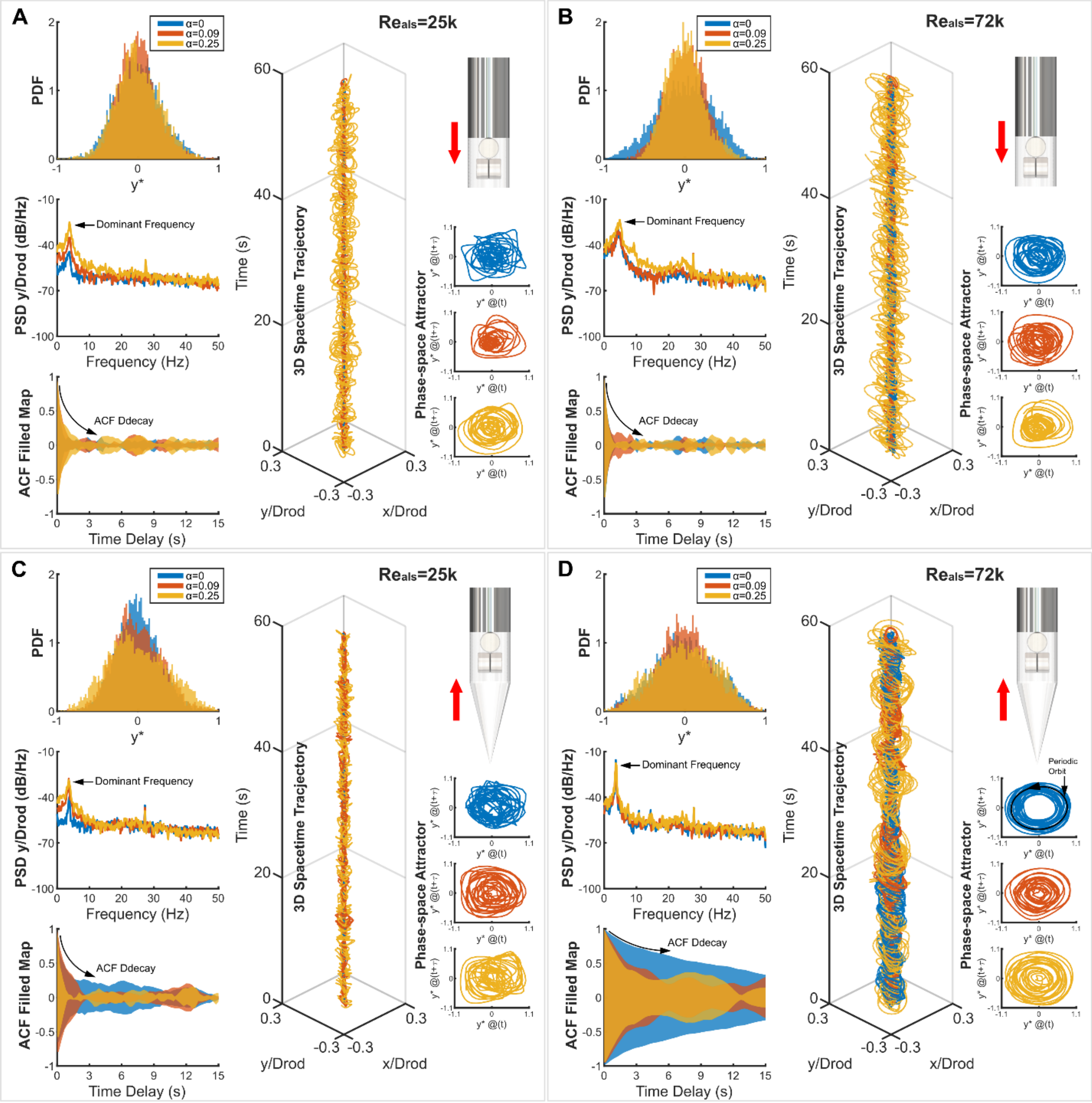

Fig. S3. Rod vibration dynamics across two-phase flow regimes and boundary conditions/geometry configurations. (**A**–**B**) Clamped-free blunt-tip dynamics. (**C**–**D**) Free-clamped cone-tip dynamics.

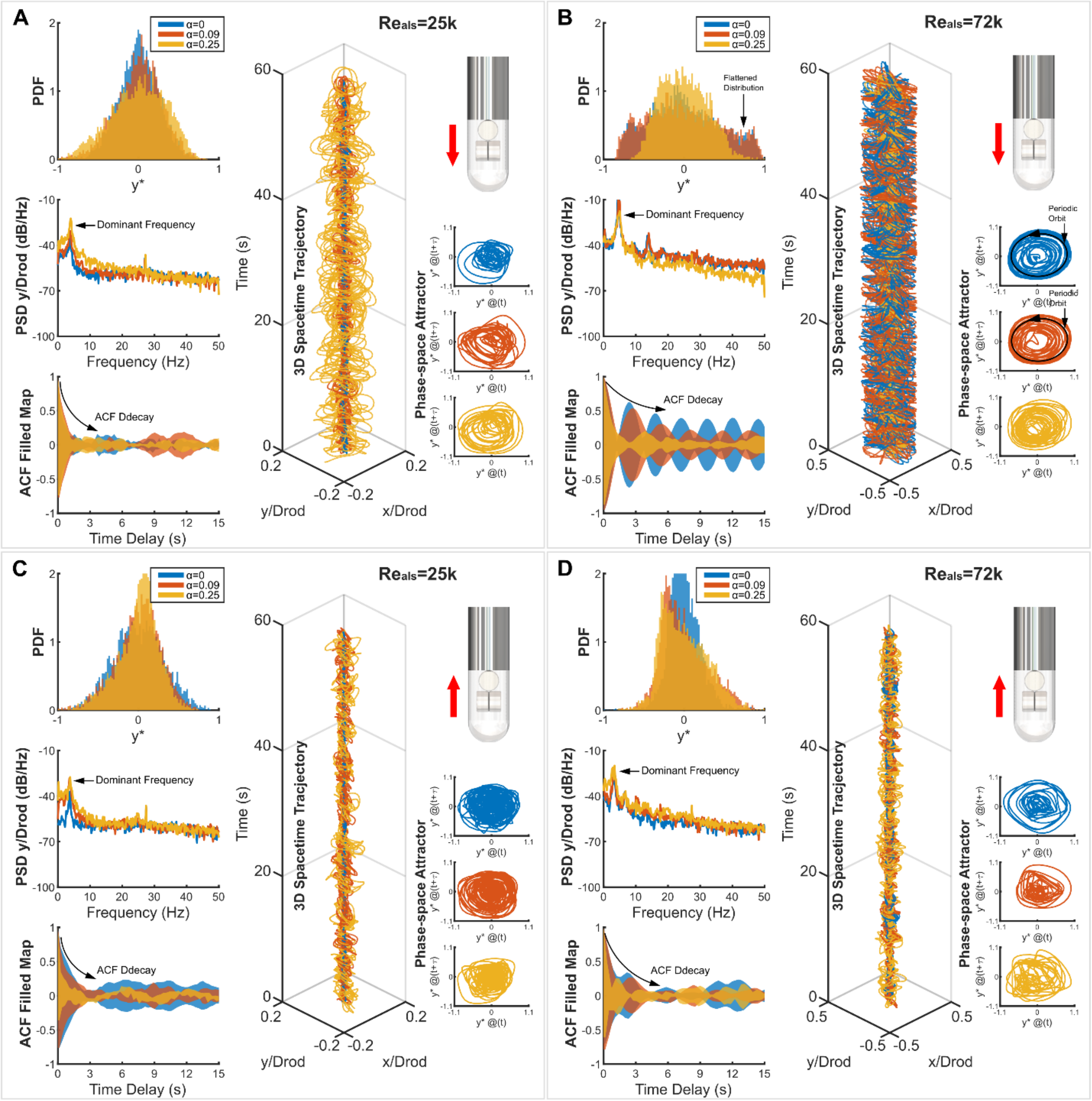

Fig. S4. Rod vibration dynamics across two-phase flow regimes and boundary conditions for the hemispherical tip geometry. (**A**–**B**) Clamped-free dynamics. (**C**–**D**) Free-clamped dynamics.

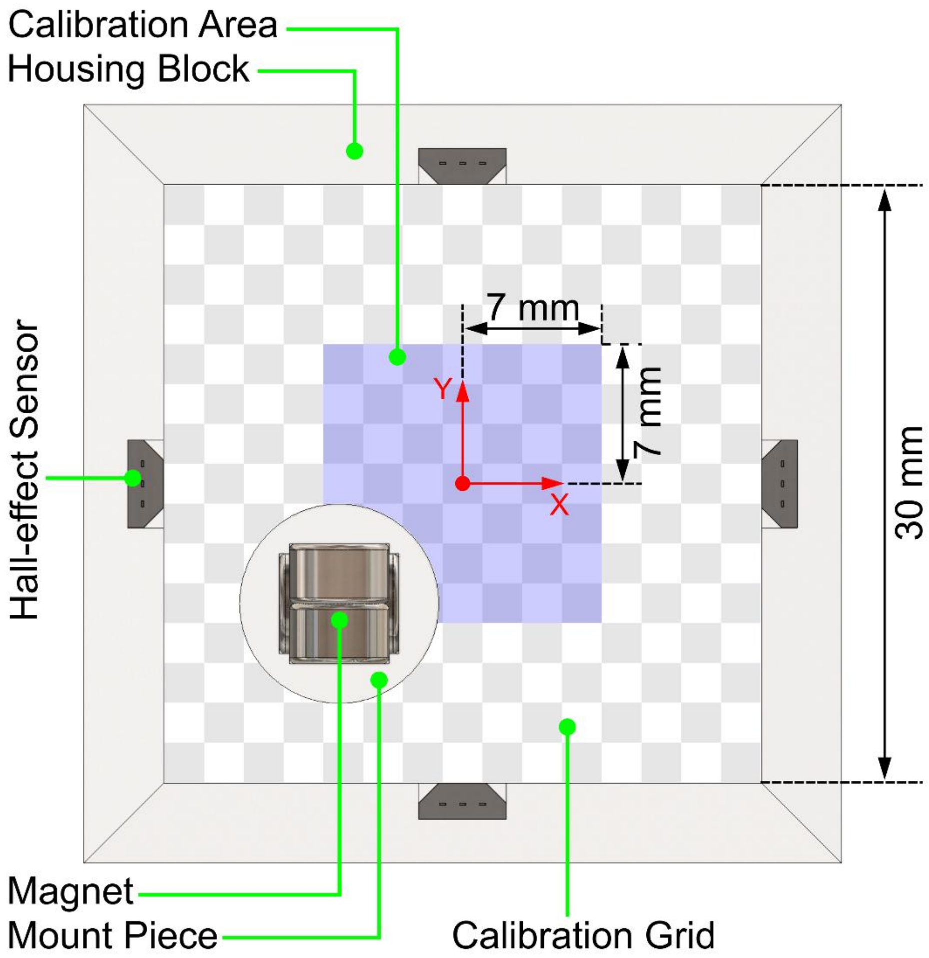

Fig. S5. Schematic of the pre-experiment calibration setup for the Hall-effect based toolkit.

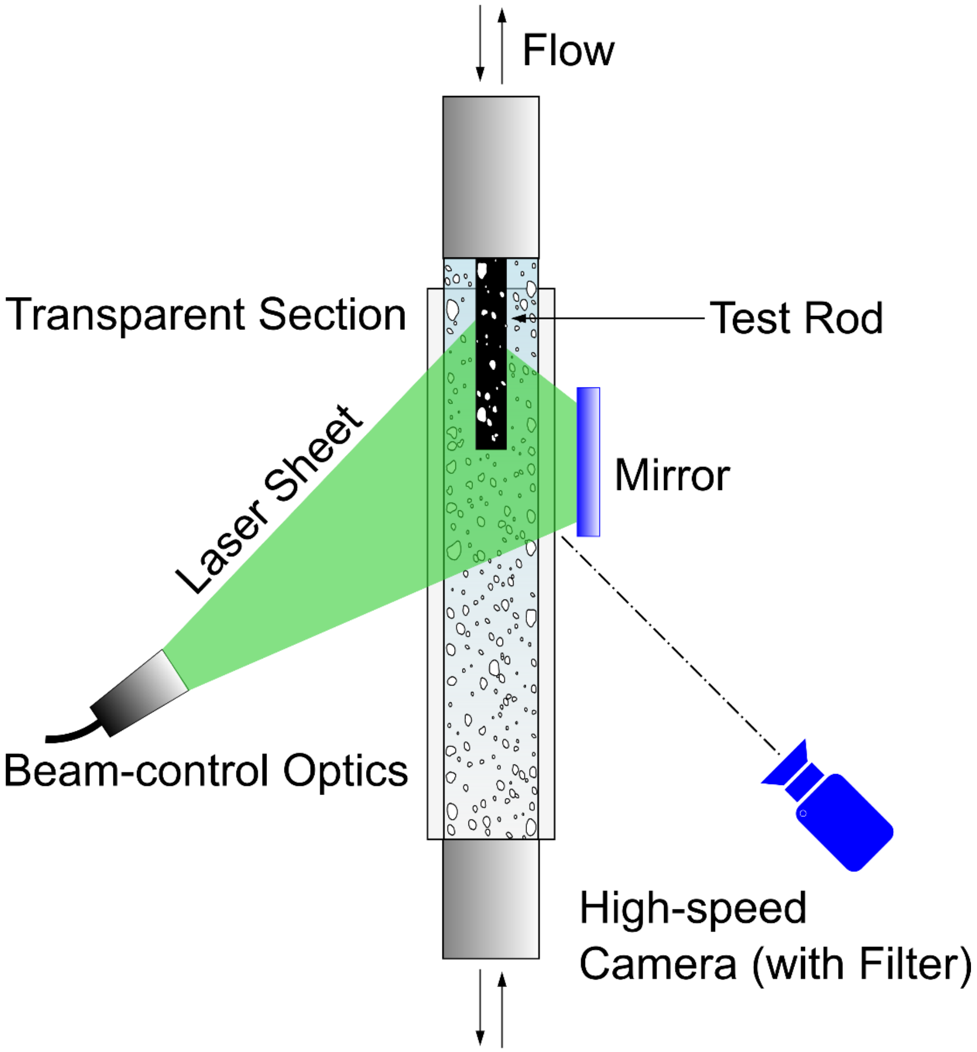

Fig. S6. Schematic of the experimental setup for flow field measurements using particle image velocimetry with laser-induced fluorescence (PIV-LIF).

Movie S1. (separate file)
30 s recording for the two-phase flow pattern – hemispherical tip geometry; clamped-free configuration at Re_{als}=72k, α=0.05. Movie frame rate: 30 fps; recording rate: 6000 fps.

Movie S2. (separate file)
30 s recording for the two-phase flow pattern – hemispherical tip geometry; free-clamped configuration at Re_{als}=72k, α=0.05. Movie frame rate: 30 fps; recording rate: 6000 fps.

Movie S3. (separate file)
30 s recording for the two-phase flow pattern – hemispherical tip geometry; clamped-free configuration at Re_{als}=25k, α=0.12. Movie frame rate: 30 fps; recording rate: 6000 fps.

Movie S4. (separate file)
30 s recording for the two-phase flow pattern – hemispherical tip geometry; free-clamped configuration at Re_{als}=25k, α=0.12. Movie frame rate: 30 fps; recording rate: 6000 fps.

Movie S5. (separate file)
30 s recording for the two-phase flow pattern – hemispherical tip geometry; clamped-free configuration at Re_{als}=72k, α=0.25. Movie frame rate: 30 fps; recording rate: 6000 fps.

Movie S6. (separate file)
30 s recording for the two-phase flow pattern – hemispherical tip geometry; free-clamped configuration at Re_{als}=72k, α=0.25. Movie frame rate: 30 fps; recording rate: 6000 fps.

Movie S7. (separate file)
30 s recording for the two-phase flow pattern – hemispherical tip geometry; clamped-free configuration at Re_{als}=25k, α=0.5. Movie frame rate: 30 fps; recording rate: 6000 fps.

Movie S8. (separate file)
30 s recording for the two-phase flow pattern – hemispherical tip geometry; free-clamped configuration at Re_{als}=25k, α=0.5. Movie frame rate: 30 fps; recording rate: 6000 fps.

Data S1. (separate file)
Measured two-phase flow-induced-vibration data for the cantilevered rod.
